# Comparison of mortality and outcomes of four respiratory viruses in the intensive care unit: a multicenter retrospective study

**DOI:** 10.1038/s41598-024-55378-x

**Published:** 2024-03-20

**Authors:** Baptiste Grangier, Charles-Hervé Vacheron, Donatien De Marignan, Jean-Sebastien Casalegno, Sandrine Couray-Targe, Audrey Bestion, Florence Ader, Jean-Christophe Richard, Emilie Frobert, Laurent Argaud, Thomas Rimmele, Anne-Claire Lukaszewicz, Frédéric Aubrun, Frédéric Dailler, Jean-Luc Fellahi, Julien Bohe, Vincent Piriou, Bernard Allaouchiche, Arnaud Friggeri, Florent Wallet, The Lyon Sud COVID-19 ICU, Fabrice Thiolliere, Emilie Joffredo, Lucille Jay, Marie Darien, Jean-Stéphane David, Charlotte Cerruti, Maxime Lecocq, Guillaume Izaute, Thomas Collenot, Olivia Vassal

**Affiliations:** 1 Service de Médecine Intensive Réanimation, Hôpital Lyon SUD, 415 chemin du grand Revoyet, 69495 Pierre-Bénite, France; 2 Service de Biostatistique - Bio-informatique, Pôle Santé Publique, Hospices Civils de Lyon, Lyon, France; 3 Laboratoire de Virologie, Institut des Agents Infectieux (IAI), Hospices Civils de Lyon, Lyon, France; 4 Centre International de Recherche en Infectiologie (CIRI), INSERM U1111, Team VirPatH, ENS Lyon, Claude Bernard Lyon 1 University, Lyon, France; 5 Pôle de Santé Publique, Département d’Information Médicale, Hôpital Edouard Herriot, Hospices Civils de Lyon, Lyon, France; 6 Service de Maladies Infectieuses et Tropicales, Hôpital de la Croix Rousse, Hospices Civils de Lyon, Lyon, France; 7 Centre International de Recherche en Infectiologie (CIRI), INSERM U1111, CNRS UMR5308, ENS Lyon, Claude Bernard Lyon 1 University, Lyon, France; 8 Service de Médecine Intensive Réanimation, Hôpital De La Croix Rousse, Hospices Civils de Lyon, Lyon, France; 9 CNRS, Inserm, CREATIS UMR 5220, U1206, Université de Lyon, Claude Bernard Lyon 1 university, INSA-Lyon, UJM-Saint Etienne, Lyon, France; 10 Service de Médecine Intensive Réanimation, Hôpital Édouard Herriot, Hospices Civils de Lyon, Lyon, France; 11 Service d’Anesthésie Réanimation, Hôpital Édouard Herriot, Hospices Civils de Lyon, Lyon, France; 12 Service d’Anesthésie Réanimation, Hôpital de la Croix Rousse, Hospices Civils de Lyon, Lyon, France; 13 Service d’Anesthésie Réanimation, Hôpital Pierre Wertheimer, Hospices Civils de Lyon, Bron, France; 14 Service d’Anesthésie Réanimation, Hôpital Louis Pradel, Hospices Civils de Lyon, Bron, France; 15 RESHAPE Research on Healthcare Performance, U1290, Claude Bernard Lyon 1 university, Lyon, France; 16 Pulmonary and Cardiovascular Aggression in Sepsis (APCSe), Université de Lyon, VetAgro Sup, Campus Vétérinaire de Lyon, UPSP 2016.A101, Marcy l’Étoile, France

**Keywords:** CARV, SARS COV 2, Influenza, RSV, ARDS, Epidemiology, Outcomes research, Infectious diseases, Influenza virus, Viral infection, Respiratory distress syndrome

## Abstract

This retrospective study aimed to compare the mortality and burden of respiratory syncytial virus (RSV group), SARS-CoV-2 (COVID-19 group), non-H1N1 (Seasonal influenza group) and H1N1 influenza (H1N1 group) in adult patients admitted to intensive care unit (ICU) with respiratory failure. A total of 807 patients were included. Mortality was compared between the four following groups: RSV, COVID-19, seasonal influenza, and H1N1 groups. Patients in the RSV group had significantly more comorbidities than the other patients. At admission, patients in the COVID-19 group were significantly less severe than the others according to the simplified acute physiology score-2 (SAPS-II) and sepsis-related organ failure assessment (SOFA) scores. Using competing risk regression, COVID-19 (sHR = 1.61; 95% CI 1.10; 2.36) and H1N1 (sHR = 1.87; 95% CI 1.20; 2.93) were associated with a statistically significant higher mortality while seasonal influenza was not (sHR = 0.93; 95% CI 0.65; 1.31), when compared to RSV. Despite occurring in more severe patients, RSV and seasonal influenza group appear to be associated with a more favorable outcome than COVID-19 and H1N1 groups.

## Introduction

Respiratory Syncytial Virus (RSV) is a single stranded RNA virus, which was described for the first time over 60 years ago^1^. Its burden in the pediatric population is well known as it is the major pathogen involved in acute lower respiratory infection (ALRI) such as bronchiolitis. Estimations based on several studies assume that RSV is responsible for almost 33 million ALRI and more than 100.000 deaths worldwide^2,3^.

However, data regarding the burden of RSV in adults, particularly in the intensive care unit (ICU) setting, remain relatively scarce due to the absence of specific clinical features in this population compared to children. Moreover, the development of specific tools for RSV identification in respiratory samples is relatively recent, further explaining the scarcity of data in adults. What has been shown is that, in elderly and high-risk adults, the virus affects about 1.5 million patients each year in industrialized countries and leads to hospitalization in 14.5% of the cases^4^. Moreover, a study reported that, in adults hospitalized with confirmed RSV infection, 57.8% were diagnosed with RSV-related pneumonia and 20.1% were admitted to an ICU; overall, the mortality rate was 10.7%^5^. RSV was also identified in 10% of respiratory samples from patients admitted to an ICU for ALRI^6^. Nevertheless, the characteristics and outcomes of adult patients admitted to ICU for RSV-related ALRI are not well described.

The prevalence of acute respiratory failure caused by community-acquired respiratory viruses (CARVs) has long been underestimated, although its economic burden has been suggested by some authors^7,8^. The two pandemics of the past decades, caused first by A(H1N1)pdm09 and then SARS-Cov-2, have reminded however, that respiratory viruses represent a major public healthcare concern^8–10^.

Data comparing the outcomes of patients infected by RSV or other CARV in the ICU are scarce.

The objective of the present study was to compare the 90-day survival and burden between the four following groups (RSV, COVID-19, Seasonal influenza and H1N1).

## Methods

### Study design

We conducted a retrospective study including patients with RSV, SARS-CoV-2 and influenza infections (distinguishing A(H1N1)pdm09 from other A and B influenza viruses) who were admitted to 6 ICU of the Lyon teaching hospital (*Hospices Civils de Lyon*, France).

This study was conducted in accordance with the amended Declaration of Helsinki and was approved by the institutional ethics committee (scientific and ethical committee of the *Hospices Civils de Lyon*, CSE-HCL, reference N° 21-505). It reviewed that our study was in strict compliance with the French reference methodology MR-004 established by the French national data protection commission (*Commission Nationale de l’Informatique et des Libertés*, CNIL, reference 21_5505). Informed consent was obtained from all subjects and/or their legal guardian(s). In accordance with French law, all patients received an information letter by post; if no objection to the use of their data was received within 30 days, the patients were enrolled. Data has been fully anonymized. No patient aged under 18 years old was included.

### Patients

All adult patients hospitalized in one of the 6 ICU between January 6, 2011 and December 28, 2018 with respiratory failure and a diagnosis of RSV infection (antigen testing, or positive RT-PCR from either bronchoalveolar lavage or nasopharyngeal specimen) were included in the RSV group. All adult patients hospitalized in one of the ICU between February 27, 2020 and April 22, 2020 with respiratory failure and a PCR-confirmed diagnosis of SARS-CoV-2 infection according to WHO interim guidance (positive RT-PCR from bronchoalveolar lavage or nasopharyngeal specimen) were included in the COVID-19 group. Given the fact that this period corresponded to the first wave in France, the circulating variants were likely to be mainly pre-VOC -alpha as it was identified in England at the end of 2020. All patients hospitalized in one of the ICU between November 1, 2015 and April 30, 2019 with respiratory failure and a PCR-confirmed diagnosis of influenza according to WHO interim guidance (positive RT-PCR from bronchoalveolar lavage or nasopharyngeal specimen) were included in the influenza group. The latter group was then split into two groups: All A serotypes (mainly H3N2) except H1N1pdm09 and all B lineages (yamagata and Victoria mainly) were included in the seasonal influenza group and A serotype (H1N1)pdm09 in the H1N1 influenza group. This selection was chosen to explore differences between ‘ancient’ CARV (RSV and seasonal influenza group), former pandemic virus now seasonal (H1N1 group) and pandemic virus (COVID-19 group). Respiratory failure was defined by the need for High-Flow Nasal Oxygen therapy (HFNO), non-invasive ventilation (NIV), or invasive mechanical ventilation (MV). Follow-up was completed on June 4, 2020 for the 4 groups. The patients in the COVID-19 and influenza groups were originally included in a previous study by our group^9^.

### Primary and secondary endpoints

The primary endpoint was survival analysis according to the type of viral infection (RSV, COVID-19, seasonal influenza, and H1N1) in patients with respiratory failure admitted to an ICU.

The secondary endpoints were related to morbidity: ICU length of stay, ICU-related complications (infection with ventilator-associated pneumonia [VAP], thromboembolism with occurrence of pulmonary embolism [PE] or deep vein thrombosis [DVT]), organ support and severity during the ICU stay (kidney failure with need for renal replacement therapy [RRT], hemodynamics with need for norepinephrine and duration of use, neurological failure according to Glasgow Coma Scale [GCS]), Sepsis-related organ failure assessment (SOFA) at Day 1, 7, 14, and SOFA_RANK_ for the first14 days^10^.

We also focused on respiratory failure by collecting data on respiratory support modalities (duration of HFNO, NIV or IMV, use of prone positioning, need for extra corporeal membrane oxygenation [ECMO]).

Baseline clinical and laboratory characteristics including clinical presentation were compared between groups. The condition “young and previously healthy”, which was recently reported in a study, was also used herein to allow comparison^11^. All variables are detailed in the supplementary information.

### Data collection

All the data analyzed in the present study were recorded on an ongoing basis in the ICU electronic health record database (IntelliSpace Critical Care and Anesthesia-ICCA; Koninklijke Philips N.V.; Amsterdam, The Netherlands) and were retrospectively collected from the reporting database. Data were obtained from the reporting database using SQL server manager studio (SSMS, v18.5. Microsoft Inc., Redmond, W.A, USA). The methods for data collection and quality control have been previously described^9^. Methods used for detection of the 4 different viruses are detailed in the supplementary information.

### Statistical analysis

Descriptive statistics were performed using median [interquartile range, IQR], and frequency (percentage) for quantitative and qualitative variables, respectively. Differences between groups were estimated using Wilcoxon Rank Sum Test for quantitative variables, and Chi-square test or Fisher’s exact test when applicable for qualitative variables. If a heterogeneity between groups was detected, a two-by-two comparison was performed to detect the group differences. The statistical threshold for two-by-two comparisons was set at 0.01.

For the survival analysis, a competing risk regression was performed, with a methodology previously described (keeping them alive approach)^12^. The 59 missing variables (< 3% per variable) were imputed using multivariate imputation by chained equations.

The following covariates, which were found to be associated with mortality in the literature, were selected for adjustment (see Supplementary table 1): age, sex, BMI, SOFA at day 1, simplified acute physiology score-II (SAPS-II), cancer, asthma/chronic obstructive pulmonary disease (COPD), diabetes, chronic kidney disease, immunosuppressive condition, and myocardial infarction^13–16^.

The competing risk regression was then used to estimate the independent effect of each virus group on mortality, adjusted on pre-specified confounding variables.

Results are expressed in adjusted sub hazard ratios (sHR) associated with their 95% CI.

Statistical significance for the p value was set at 0.05. Statistical analyses were performed using the package survival from R software V 3.6.3.

### Ethics approval and consent to publications

This study was conducted in accordance with the amended Declaration of Helsinki and was approved by the institutional ethics committee (Scientific and Ethical Committee of the *Hospices Civils de LYON*, CSE-HCL, under reference N° 21-505). It reviewed that the study was in strict compliance with the French reference methodology MR-004 established by the French national data protection commission (*Commission nationale de l’informatique et des libertés*, CNIL, under reference N° 21_5505). Non-opposition for data use was obtained from the patients. All patients received an information letter by post; if no objection was received within 30 days, the patients were enrolled in accordance with French law. Data has been fully anonymized. No patients under 18 years old were enrolled.

## Results

### Patients

A total of 832 patients were screened and 807 were finally included (supplementary Information). In the RSV group, 56% of the positive tests were performed on nasopharyngeal specimens and 44% on bronchoalveolar fluid.

Patients admitted for RSV had more comorbidities according to Charlson Comorbidity Index (CCI). More specifically, they were more likely to have a history of myocardial infarction, congestive heart failure, active solid tumor or metastatic cancer, history of respiratory disease (asthma, COPD), and were more likely to have been hospitalized in the past 12 months (Table 1).

**Table 1:** Characteristics of critically ill patients at admission in the intensive care unit according in the 4 groups.

	Total n = 807	RSV n = 151	COVID-19 n = 332	Seasonal influenza n = 258	H1N1 influenza n = 66	*P*
Age (years)	70 [60; 79]	72 [60; 79]	68 [59; 77]	72 [62; 81]	66 [55; 74]	**0.001**
Sex (male)	501 (62)	89 (59)	239 (72)	127 (49)	46 (67)	** < 0.001**
BMI (Kg.m^-2^)	27 [23; 30]	26 [23; 30]	27 [24; 31]	26 [22; 31]	25 [22; 28]	** < 0.001**
CCI	2 [1; 4]	5 [3; 7]	1 [0; 2]	2 [1; 4]	2 [1; 3]	** < 0.001**
Young and previously healthy	39 (5)	4 (3)	21 (6)	8 (3)	6 (9)	0.094
Arterial hypertension	421 (52)	62 (41)	181 (55)	142 (55)	36 (54)	**0.024**
Myocardial infarction	122 (15)	38 (25)	30 (9)	43 (17)	11 (17)	** < 0.001**
Congestive heart failure	180 (22)	70 (46)	35 (11)	62 (24)	13 (20)	** < 0.001**
Arteriopathy of lower limbs	67 (8)	11 (7)	19 (6)	29 (11)	8 (12)	0.065
Cerebral stroke	37 (5)	4 (3)	28 (8)	22 (8)	7 (11)	**0.046**
Hemiplegia	27 (3)	3 (2)	6 (2)	15 (6)	3 (4)	**0.038**
Dementia	35 (4)	3 (2)	13 (4)	16 (6)	3 (4)	0.232
Cirrhosis	28 (3)	6 (4)	7 (2)	12 (5)	3 (4)	0.283
Chronic kidney disease	47 (6)	14 (9)	11 (3)	14 (5)	8 (12)	**0.007**
Chronic dialysis	37 (5)	9 (6)	8 (2)	12 (5)	8 (12)	**0.007**
Immunosuppressive treatment	114 (14)	28 (18)	18 (5)	54 (21)	14 (21)	** < 0.001**
Hospital stay within 12 months	280 (35)	86 (58)	55 (17)	113 (44)	26 (39)	** < 0.001**
Diabetes	215 (27)	45 (29)	92 (28)	64 (25)	14 (21)	0.493
COPD/asthma	226 (28)	67 (44)	36 (11)	106 (41)	17 (26)	** < 0.001**
Current or former smoker	310 (39)	69 (47)	100 (30)	108 (42)	33 (50)	** < 0.001**
Chronic alcoholism	97 (12)	13 (9)	27 (8)	40 (15)	17 (26)	** < 0.001**
Cancer in remission for more than 5 years	87 (11)	3 (2)	31 (9)	46 (18)	7 (11)	** < 0.001**
Active solid cancer	99 (12)	37 (24)	16 (5)	36 (14)	10 (15)	** < 0.001**
Metastatic active solid cancer	33 (4)	14 (9)	4 (1)	13 (5)	2 (3)	** < 0.001**
Leukemia	13 (2)	4 (3)	0 (0)	8 (3)	1 (1)	**0.003**
Lymphoma	36 (4)	4 (3)	11 (3)	15 (6)	6 (9)	0.09
SAPS-II score	43 [33; 57]	51 [39; 71]	37 [28; 68]	45 [38; 61]	51 [41; 63]	** < 0.001**
SOFA score at admission	6 [3; 10]	8 [4; 11]	4 [2; 8]	8 [5; 11]	9 [5; 12]	** < 0.001**
Fever at admission	519 (68)	48 (35)	258 (84)	165 (65)	48 (78)	** < 0.001**

Data are presented as median [IQR] or n (%).COPD, chronic obstructive pulmonary disease; SAPS-II, Simplified acute physiology score; SOFA, sepsis-related organ failure assessment. *P* value are estimated for the heterogeneity among the four groups.

Significant values are in bold.

Patients in the COVID-19 group were less frequently active or past smokers as well as chronic alcohol consumers and less often a history of active cancers. Immunosuppressive treatments were significantly less frequent in the COVID-19 group compared to the other 3 groups. Few patients (5%) were considered as young and previously healthy, with no significant difference between groups (Table 1).

At admission, patients in the COVID-19 group were significantly less severe according to the SAPS-II and SOFA scores at admission compared with seasonal influenza, RSV, and the H1N1 group, see Table 1. Fever at admission was less frequent in the RSV group (35%) compared with the H1N1 (77%), seasonal influenza (65%) and COVID-19 groups (84%, *p* < 0.001; Table 1). At admission, there were significant differences among the 4 groups regarding laboratory parameters (Table 2).

**Table 2:** Laboratory parameters at admission of critically ill patients in the 4 groups.

	RSV n = 151	COVID-19 n = 332	Seasonal influenza n = 258	H1N1 Influenza n = 66	*P*
Bilirubin (µmol/L)	10 [6; 22]	10 [7; 14]	9 [7; 15]	14 [10; 17]	0.134
Creatinine (µmol/L)	105 [73; 204]	81 [64; 118]	98 [68; 163]	102 [71; 207]	** < 0.001**
Lactic acid (mmol/L)	2.0 [1.4; 3.6]	1.6 [1.3; 2.1]	1.9 [1.3; 3.1]	1.8 [1.2; 2.5]	**0.004**
Platelets (G/L)	193 [149; 254]	227 [167; 300]	203 [133; 265]	185 [142; 217]	**0.014**
Leucocytes (G/L)	11.1 [8.0; 15.8]	7.7 [5.7; 11.0]	10.6 [7.1; 13.6]	11.2 [5.4; 15.5]	** < 0.001**
Lymphocytes (G/L)	0.8 [0.5; 1.2]	0.8 [0.6; 1.1]	0.6 [0.4; 1.0]	0.5 [0.3; 0.9]	**0.006**

Data are presented as median [IQR]. *P* value are estimated for the heterogeneity among the four groups.

Significant values are in bold.

Concerning specific therapies, 259 patients (77%) admitted for influenza (seasonal and H1N1) received oseltamivir, 11 patients (3%) in the COVID-19 group received remdesivir, 69 (14%) received hydroxychloroquine, 14 (4%) received ritonavir and 4 patients (3%) in the RSV group received ribavirine. Overall, 240/807 (30%) patients were treated with corticoids during their ICU stay, with no statistical difference between groups.

### Survival analysis

In comparison with the RSV group, the COVID-19 group (sHR 1.61 CI.95 [1.10–2.36] *p* = 0.014) and H1N1 group (sHR 1.87 CI.95 [1.20–2.93] *p* < 0.001) were associated with a higher mortality. Conversely, seasonal influenza was not associated with a higher mortality compared to RSV (sHR 0.93 CI.95 [0.65–1.31] *p* = 0.67; supplementary table 1). For patients aged 65 years and older, mortality in the ICU was 35% in the RSV group, 50% in the H1N1 group, 28% in the seasonal influenza group, and 37% in the COVID-19 group (*p* = 0.064).

### Patient severity during ICU stay

Length of stay (LOS) in ICU for patients in the H1N1 and COVID-19 group were significantly longer than in the 2 others groups, see Table 3.

**Table 3:** Outcomes of critically ill patients in the 4 groups.

	Total n = 807	RSV n = 151	COVID-19 n = 332	Seasonal influenza n = 258	H1N1 influenza n = 66	*P*
Length of stay in ICU (days)	8 [4; 20]	7 [4; 15]	11 [4; 25]	6 [3; 13]	11 [6; 24]	** < 0.001**
Mortality in ICU	229 (28%)	44 (29%)	92 (28%)	64 (25%)	29 (44%)	**0.022**
SOFA at Day 1	6 [3; 10]	8 [4; 11]	4 [2; 8]	8 [5; 11]	9 [5; 12]	** < 0.001**
SOFA at Day 7	7 [4; 11]	7 [3; 9]	9 [5; 11]	5 [3; 8]	8 [4; 12]	** < 0.001**
SOFA at Day 14	7 [4; 11]	5 [2; 10]	8 [4; 11]	4 [3; 8]	9 [4; 13]	** < 0.001**
SOFA_RANK_ from day 1 to 14		− 39 [− 81; 5]	0 [-12; 70]	− 15 [− 58; 19]	10 [− 32; 92]	** < 0.001**

Data are presented as median [IQR] or n (%). SOFA, sepsis-related organ failure assessment. *P* value are estimated for the heterogeneity among the four groups.

Significant values are in bold.

Regarding organ failure and required support, patients in the H1N1 group required more frequently invasive MV (80%, *p* < 0.001) but its duration in the COVID-19 group was significantly longer, see Table 4. The rate of prone positioning was also higher in the COVID-19 group, see Table 4. Patients in the H1N1 and COVID-19 groups received more often neuromuscular blockade agents, see Table 4. Regarding non-invasive respiratory support, HFNO was more frequently used in the COVID-19 group and for a longer period, see Table 4. Patients in the RSV group received bronchodilators in a significantly higher proportion (intravenous and double aerosol therapy; see Table 4). Incidence of DVT and PE was higher in the COVID-19 group, see Table 4. In terms of organ support and severity, RRT was more frequent in the H1N1 group, see Table 4. There was no significant difference in vasopressor requirement between groups but when needed, it was used during a significantly longer period in the COVID-19 group, see Table 4. The evolution profile of the patients in the 4 groups differed significantly as highlighted by a negative SOFA_RANK_ on the 14 first days for the RSV and seasonal influenza groups and a positive SOFA_RANK_ the COVID-19 and H1N1 groups (Table 3).

**Table 4:** Organ dysfunction of critically ill patients in the 4 groups.

	Total n = 807	RSV group n = 151	Covid-19 n = 332	Seasonal influenza n = 258	H1N1 influenza n = 66	*P*
Invasive mechanical ventilation (IMV)	476 (59)	98 (65)	181 (55)	144 (56)	55 (80)	** < 0.001**
IMV duration (days)	10 [4; 21]	4 [0; 8]	19 [12; 31]	8 [5; 15]	12 [5; 22]	** < 0.001**
Neuromuscular blockade	363 (45)	67 (44)	178 (54)	80 (31)	38 (57)	** < 0.001**
Duration of neuromuscular blockade (days)	2 [1; 5]	0 [0; 2]	5 [2; 9]	1 [1; 3]	2 [1; 5]	** < 0.001**
Prone positioning	242 (30)	28 (18)	150 (45)	41 (16)	23 (35)	** < 0.001**
HFNO	324 (40)	34 (22)	202 (61)	64 (25)	24 (36)	** < 0.001**
Duration of HFNO (days)	3 [2–5]	2 [2–4]	3 [2–6]	2 [1–4]	2 [1–2]	** < 0.001**
NIV	399 (49)	137 (90)	87 (26)	146 (57)	29 (44)	** < 0.001**
Duration of NIV (days)	3 [1; 5]	3 [2; 4]	2 [1; 4]	2 [1; 5]	4 [2; 4]	0.104
ECMO	17 (2)	1 (1)	9 (3)	4 (2)	5 (8)	0.057
Beta agonists aerosols	324 (40)	89 (59)	61 (18)	131 (54)	35 (53)	** < 0.001**
Duration (days)	6 [3; 11]	7 [5; 12]	3 [1; 5]	6 [3; 9]	10 [4; 15]	** < 0.001**
anticholinergics aerosols	154 (19)	59 (39)	26 (8)	57 (22)	12 (18)	** < 0.001**
Vasopressors	471 (58)	95 (63)	184 (55)	146 (57)	46 (70)	0.098
Duration of vasopressors (days)	5 [2; 10]	3 [0; 5]	10 [5; 18]	4 [2; 6]	5 [3; 10]	** < 0.001**
RRT	152 (19)	23 (15)	65 (20)	38 (15)	26 (39)	** < 0.001**
VAP	144 (18)	22 (15)	78 (23)	29 (11)	15 (23)	** < 0.001**
Venous thrombo embolic complications	79 (10)	7 (5)	53 (16)	15 (6)	4 (6)	** < 0.001**

Data are presented as median [IQR] n (%). *P* value are estimated for the heterogeneity among the four groups.

Significant values are in bold.

## Discussion

In this large retrospective cohort study, adjusted mortality at day-90 was significantly higher in the H1N1 and COVID-19 groups than for those in the RSV and seasonal influenza groups. At admission, the clinical presentation and laboratory parameters differed according to the infecting virus. Patients in the RSV group had more comorbidities, especially respiratory, and those in the COVID-19 group were the least severe at admission. The evolution of patients also differed as patients in the COVID-19 and H1N1 group present with more severe organ failure, especially respiratory, during the ICU stay. This strongly suggests that RSV and seasonal influenza intrinsically induce less severe pneumonia in more comorbid patients, as previously suggested^9^.

The present study is one of the largest comparing RSV with other CARV in an adult ICU population and the first to include SARS-CoV-2. Until now, only a few studies have compared CARV pneumonia in non-critical care patients^17,18^ and their results corroborate the present findings. One recent study comparing the outcomes of patients admitted to the ICU for respiratory failure related to RSV and influenza^19^ found an unadjusted ICU mortality rate slightly lower (19.4%) than that found herein. However, the authors reported that adjusted mortality did not differ significantly between the RSV and influenza groups (adjusted OR 0.80. CI.95 [0.49–1.30], *p* = 0.37), a result in line with those reported herein.

One explanation for the higher mortality rate observed herein compared to that of Coussement et al. could be that they included all patients with a positive PCR for RSV or influenza, with about a third of patients in each group receiving only low flow oxygen, whereas the present cohort included only patients with respiratory failure (i.e. need for MV, HFNO, or NIV). Moreover, 17 and 21% (RSV and influenza groups, respectively) of the patients in the study by Coussement et al. were admitted to the ICU for another reason than respiratory failure.

Further corroborating the present results, another study reported that COVID-19 and H1N1 patients had a higher 30-day mortality rate, longer ICU LOS, and longer duration of IMV although being less frail and less severe at admission when compared to an ICU population affected by other viruses including RSV and seasonal influenza^20,21^.

The present findings demonstrate the major burden of RSV in ICU with similar mortality and clinical outcomes than seasonal influenza. Thus, RSV should be systematically considered in patients admitted to the ICU for acute respiratory failure and this search should be carried out for a longer period than influenza, given the physiopathology of RSV^22^. Moreover, the present findings indicate that the clinical presentation differs according to the virus, which could allow a better identification of patients at risk. Conversely to patients affected by SARS-CoV-2 and A(H1N1)pdm09 influenza, patients with RSV are more severe at admission because of their comorbidities but have more favorable outcomes. These results suggest a higher intrinsic virulence of the most recent respiratory viruses. Since SARS-CoV-2 first appeared at the end of 2019, the world has faced successive outbreaks of the virus, which will likely stay endemic like RSV or influenza^23–26^. The successive variants have demonstrated a change in the course of the disease, with a trend toward a lower overall mortality, but an increase in the burden for patients with comorbidities, especially respiratory^27^ and immunocompromised ones^28,29^.

None of the specific treatment received by all the patients was associated with survival improvement. Of note, ribavirin, which is the only specific therapeutic against RSV currently available but whose benefit remains controversial^30^, was rarely given in the RSV group. While new drugs for RSV treatment^31–33^ are currently in development, management remains mainly supportive at this time. Although nirsevimab was recently proposed for at risk children^34^, no preventive strategy is available for adults. In light of the present findings regarding the burden of RSV in ICU adults, the need for new antiviral drugs and vaccines targeting RSV appears paramount.

The present study has limitations. First, data were collected retrospectively. We tried to minimize this bias by paying particular attention to the quality of the data collected, checking the diagnostic accuracy for all patients, and using the same queries and methodology for each group. Another limitation concerns the comparison of the four viruses over different periods, which could underline potential differences in patient management. However, all data were obtained from ICUs of the same teaching hospital that apply similar management approaches. Moreover, this bias is likely to concern mainly patients affected by RSV before 2013 (25/151), when the publication by Guerin et al. demonstrated the benefits of prone positioning in ARDS^35^. Indeed, other therapeutics (protective ventilation^36,37^, NMBA^38,39^) relied on studies older than the data collection periods considered herein. In the same way, the relatively low proportion of COVID-19 patients treated by corticoids could be a limitation. This is likely due to the fact that the inclusion of this group was made during the first wave of the pandemic, before the publication of high quality data supporting the use of corticoids in severe COVID-19^40^.

The choice was made to select potential confounders based on prior knowledge and not on univariate analysis, as the latter practice has been shown to be strongly biased^13–16^. The variables included in the model were thus chosen before any statistical analysis, explaining the non-significant differences of certain confounders entered in the model.

The major strengths of this work are its large sample size, the selection of patients with ARF admitted to the ICU, and the inclusion of four major CARV in a multicenter design.

In conclusion, the present study highlights important differences in patient characteristics according to the virus involved. RSV and seasonal influenza are associated with more severe patients at admission because of their comorbidities but a more favorable outcome, in contrast to patients in the COVID-19 and H1N1 group.

### Supplementary Information

Supplementary Information.

## Data Availability

FW takes responsibility for the content of the manuscript, including the data and analysis. Data are available to reviewers upon request to the corresponding author.

## Abbreviations

ALRI: Acute lower respiratory infection
ARDS: Acute respiratory distress syndrome
ARF: Acute respiratory failure
BMI: Body mass index
CARV: Community acquired respiratory viruses
95% CI: 95% Confidence interval
COPD: Chronic obstructive pulmonary disease
CT: Computed tomography
DVT: Deep vein thrombosis
ECMO: Extra corporeal membrane oxygenation
FiO2: Fraction of inspired oxygen
HFNO: High-flow Nasal oxygen therapy
sHR: Sub Hazard ratio
GCS: Glasgow Coma Scale
ICU: Intensive care unit
LOS: Length of stay
IMV: Invasive mechanical ventilation
NIV: Non-invasive ventilation
NMBA: Neuromuscular blocking agent
PaO2: Partial pressure of oxygen in blood
PE: Pulmonary embolism
PEEP: Positive end expiratory pressure
PCR: Polymerase chain reaction
RRT: Renal replacement therapy
RT-PCR: Reverse-transcriptase polymerase chain reaction
SAPS-II: Simplified acute physiology score
SOFA: Sepsis-related organ failure assessment
RSV: Respiratory syncytial virus
VAP: Ventilator-associated pneumonia
