# Supplementary material for: Comparison of mortality and outcomes of four respiratory viruses in the intensive care unit: a multicenter retrospective study
Source: Sci Rep. 2024 Mar 20;14:6690. doi: 10.1038/s41598-024-55378-x (PMC10954612; doi:10.1038/s41598-024-55378-x)
Supplement: Supplementary file 1 — Supplementary Information. [file 41598_2024_55378_MOESM1_ESM.docx]

Supplementary Information

[**Flowchart of patients included in the study according to virus group** 2](#_Toc153818478)

[**Detailed methodology for data mining** 3](#_Toc153818479)

[**Data collection and definition** 4](#_Toc153818480)

[**Detailed methods used for the detection of RSV, influenza, and SARS-CoV-2** 8](#_Toc153818481)

[**Supplementary Table 1** 9](#_Toc153818482)

**Flowchart of included patients in the COVID and Influenza group**

832 patients screened

RSV

185 patients

Influenza

325 patients

COVID-19

350 patients

RSV

151 patients

included

34 patients

Excluded =

No respiratory failure

324 patients

included

1 patient

Refused to participate

Seasonal influenza

258 patients

H1N1 influenza

66 patients

COVID-19

332 patients

included

18 patients

Refused to participate

**Detailed methodology for data mining**

The COVID-19 database was prospectively obtained since the first patient was admitted to the institution. The influenza database was retrospectively constructed with the use of the national coding system using the international classification for disease v11 (ICD-11). Codes for influenza were retrieved for the patients admitted to the ICU between November 1, 2015 and April 30, 2019.

The following ICD-11 codes were used J10.0, J10.1, J10.8, J09.0, J11.0, J11.1, and J11.8

Two investigators (DDM, FW) checked all the patient records individually to ensure that influenza and COVID-19 diagnoses were correct.

The RSV database was constructed by retrieving all positive RSV specimens from the virology laboratory. Then, all patients admitted to the ICU with a positive specimen ranging from five days before or after ICU admission were selected. All medical records were individually checked to ensure that admission was related to acute respiratory distress linked with RSV.

The influenza RSV and covid databases were then computed and analyzed using the same SQL queries in order to minimize bias during data recovery.

SQL queries were computed with SQL server management studio (SSMS), v18.5, Microsoft Inc., Richmond, USA using transact-SLQ language. All SQL queries where manually checked to assess their effectiveness and relevance.

All data where then quality-checked and all data out of the range or inconsistent were manually checked and corrected if needed. We used logical queries to look for any inconsistencies in the data (as an example, if a mean tidal volume is available at one time, then this patient must be declared as receiving mechanical ventilation or noninvasive ventilation. Conversely, if a patient is associated with mechanical invasive ventilation, a tidal volume must be available between intubation and extubation).

Authors (BG, DDM, AF, and FW) manually obtained the data for which exhaustiveness was insufficient with SQL queries.

**Data collection and definition**

The following variables were collected:

- Patient characteristics:
- demographic and anthropometric data,
- hospital and ICU admission date
- date of first symptoms of COVID-19 infection
- comorbidities
  - Charlson Comorbidity Index
  - Hypertension
  - ACE (Angiotensin Converting Enzyme) inhibitor/ARB (Angiotensin Receptor blockers)
  - Ischemic heart disease
  - Congestive heart disease
  - Arteriopathy of lower limbs
  - Cerebral stroke
  - Hemiplegia
  - Dementia
  - Cirrhosis
  - HIV infection
  - Immunosuppressive treatment
  - Hospital stay within 12 months
  - Diabetes and complicated diabetes§
  - COPD/Asthma
  - Chronic respiratory disease
  - Current smoker
  - Chronic ethylism
  - Pregnancy
  - Cancer in recovery for more than 5 years
  - Active solid cancer
  - Metastatic active solid cancer
  - Hemopathy
- SAPS II and SOFA scores at ICU admission and different time points
- Morbidity data:
- ICU length of stay.
- ICU related complications: thrombo-embolism event and ventilatory associated pneumonia
- respiratory and extra-respiratory organ support during ICU stay
  - use of mechanical ventilation and duration
  - use of neuromuscular blockade and duration
  - use of prone positioning and duration
  - use of PEEP level of more than 15 cmH2O and duration
  - use of HFNO and duration
  - Use of non-invasive ventilation and duration
  - Use of vasopressor and duration
  - use of RRT and duration
  - mean driving pressure during the 24 first hours after intubation if invasive ventilation
- Daily respiratory parameters assessed at Day 1, Day 3, Day 7, and Day 14
  - Proportion of patients under mechanical ventilation, neuromuscular blockade, prone positioning therapy, HFNO, NIV; Mean PaO2, FiO2, PaO2/FiO2, tidal volume (adjusted on predicted body weight), and maximal value of PEEP.
- Daily extra-respiratory parameters assessed at Day 1, Day 3, Day 7, and Day 14:
  - SOFA score, SOFA without the respiratory item, proportion of patients under vasopressors, RRT, and mean values of lactatemia, lymphocytosis, creatinine, fibrinogenemia.

Immunosuppression status was defined as steroids at any dose for more than 1 month or any other immunosuppressive treatment.

SAPS II is an ICU scoring system. Its name stands for "Simplified Acute Physiology Score II".

It is based on the following parameters: age, heart rate, systolic blood pressure, [t](https://en.wikipedia.org/wiki/Thermoregulation)emperature,

[Glasgow coma scale](https://en.wikipedia.org/wiki/Glasgow_Coma_Scale), mechanical ventilation, [PaO2](https://en.wikipedia.org/wiki/PaO2)/[FiO2](https://en.wikipedia.org/wiki/FiO2) ratio, urine output, chronic diseases,

type of admission, and some blood analysis: [blood urea nitrogen](https://en.wikipedia.org/wiki/Blood_Urea_Nitrogen), sodium, potassium,

bicarbonate, bilirubin, and white blood cell. SAPS II is generally used to define the initial severity of patients admitted to the ICU.

Sequential Organ Failure Assessment (SOFA) is another ICU scoring system generally used to

evaluate severity at a defined moment and its evolution. It is simpler to calculate than the SAPS II. It is based on 6 different scores, each for the respiratory, cardiovascular,

neurological, renal, coagulation, and hepatic systems.

**Detailed methods used for the detection of RSV, influenza, and SARS-CoV-2**

1. RSV detection

RSV was detected for the first-enrolled patients by antigenic method: Sofia RSV immunoassays (SOFIA®RSV, QUIDEL, San Diego, CA).

However, PCR detection was used for the majority of patients: MWS RSV/hMPV r-gene^®^ PCR kit (BioMérieux, Lyon, France) on the ABI PRISM^®^ 7500 system (Applied Biosystems, Foster city, CA, USA).

1. Influenza detection

From 2015 to 2018, Flu detection was performed by a semi-automated approach including genome extraction on easyMag (BioMérieux, France) followed by amplification on ABI Prism 7500 (Applied Biosystems) using the MWS r-gene^®^ Influenza A/B kit (Argène BioMérieux, France). Since 2019, the FluA/B/RSV Assay on Panther Fusion™ System (Hologic, Marlborough, MA) is used for influenza detection.

1. SARS-CoV 2 detection
2. the QS technique developed by Institut Pasteur (Paris, France);
3. the QS technique adapted on the open access channel of the automated system Panther Fusion (Hologic^®^, Marlborough, MA)
4. the assay Cobas^®^ SARS-CoV-2 was used on the Cobas^®^ 6800 system (Roche Diagnostics^®^, Bale, Switzerland)
5. The Eurobioplex SARS-CoV-2 kit (Eurobio Scientific^®^, Les Ulis, France).

**Supplementary Table 1**

Cox model – effects of group alone (model 1) or full model (model 2)

| Model 1: effects of group alone (COVID-19, seasonal influenza, H1N1 influenza, RSV) |
| --- |

|  |  | | Sub-Hazard ratio | p-value |
| --- | --- | --- | --- | --- |
| RSV |  | | Reference group |  |
| COVID-19 | 1.58 | | [1.08;2.31] | 0.01954 |
| Seasonal influenza | 0.93 | | [0.65;1.31] | 0.66688 |
| H1N1 influenza | | 1.87 | [1.20;2.93] | 0.00583 |
|  |  | |  |  |

Model 2: final model parameters

|  | Value | Missing | Sub-Hazard ratio | p |
| --- | --- | --- | --- | --- |
| Group | RSV |  | reference |  |
|  | COVID-19 |  | 1.61 [1.10;2.36] | **0.014** |
|  | Seasonal influenza |  | 0.93 [0.65;1.31] | 0.670 |
|  | H1N1  influenza |  | 1.87 [1.20;2.93] | **0.006** |
| Age per 10 years |  | 0 | 1.51 [1.34;1.70] | **<0,001** |
| Sex | Female | 0 | reference |  |
|  | Male |  | 1.37 [1.05;1.80] | **0.021** |
| BMI (per point) |  | 16 | 1.00 [0.97;1.02] | 0.677 |
| SOFA Day 1 (per point) |  | 21 | 1.08 [1.04;1.12] | **< 0.001** |
| SAPS II (per point) |  | 24 | 1.13 [1.04;1.24] | **0.006** |
| Cancer | no | 1 | Reference |  |
|  | yes |  | 1.83 [1.37;2.45] | **< 0.001** |
| Asthma/COPD | no | 1 | reference |  |
|  | yes |  | 1.25 [0.94;1.67] | 0.124 |
| Diabetes | no | 0 | Reference |  |
|  | yes |  | 0.96 [0.72;1.26] | 0.746 |
| Chronic kidney disease | no | 0 | Reference |  |
|  | yes |  | 0.86 [0.53;1.41] | 0.552 |
| Immunosuppressive condition | no | 0 | Reference |  |
|  | yes |  | 1.48 [1.05;2.10] | **0.026** |
| Myocardial infarction | no | 0 | Reference |  |
|  | yes |  | 1.10 [0.80;1.53] | 0.554 |
